# New Frontiers in Ventricular Pacing

**DOI:** 10.19102/icrm.2018.090503

**Published:** 2018-05-15

**Authors:** Jay A. Montgomery, Christopher R. Ellis

**Affiliations:** ^1^Vanderbilt Heart and Vascular Institute, Nashville, TN, USA

**Keywords:** His-bundle pacing, right ventricular apical pacing, right ventricular septal pacing

Since endocardial pacing replaced epicardial pacing in the 1960s, right ventricular (RV) apical pacing has been the default standard of care for ventricular pacing.^1^ However, several trials have revealed significant evidence of the harm caused by chronic right ventricular pacing (primarily from the RV apex).^2–4^ Primary and secondary analyses from the Mode Selection Trial in Sinus-node Dysfunction (MOST), Dual Chamber and VVI Implantable Defibrillator (DAVID), and Biventricular Versus RV Pacing in Heart Failure Patients With Atrioventricular Block (BLOCK-HF) studies show not just a binary effect but also a dose-dependent response effect with regard to reduced left ventricular function, heart failure, and even death with increased RV pacing burden.^2–4^ This dose-dependent response has been seen in observational studies as well.^5,6^ This is thought to be primarily due to interventricular dyssynchrony, and there is strong evidence that cardiac resynchronization therapy (CRT) mitigates this risk.^2^

It is in this setting that Worsnick et al. review alternatives to RV apical pacing in this issue of *The Journal of Innovations in Cardiac Rhythm Management.*^7^ As they note, there is growing evidence and enthusiasm for alternative sites for ventricular pacing, including the RV septum; the RV outflow tract; and, most recently, the His-bundle region. As they mention, several small trials of RV outflow or RV septal pacing versus RV apical pacing have shown somewhat conflicting results, with most studies indicating a shorter QRS duration with RV nonapical pacing but failing to show a clinical benefit. However, statistical power has been lacking, as only two of these trials contained more than 100 patients. In addition, there is a lack of standardization for RV septal and RV outflow lead placement positions, and it is not always completely clear to which endocardial surface a lead may be attached. With this in mind, Worsnick et al.’s discussion of methods for obtaining a true septal lead position is valuable for anyone unfamiliar with the technique.

Perhaps most interesting is the discussion of His-bundle pacing by respected authors in this field. The excitement surrounding His-bundle pacing across the electrophysiology community is growing, particularly on social media (#dontdisthehis). Randomized data for His-bundle pacing are currently lacking, but at least two randomized trials are ongoing (NCT02805465 and NCT02700425) with expected completion in 2018 and 2021, respectively. Furthermore, nonrandomized studies have suggested that His-bundle pacing gives significant benefits over RV apical pacing and is a reasonable substitute for CRT (including for resynchronization of left bundle branch block). However, whenever nonrandomized data are reported, there is an inherent publication bias toward positive results. Further considerations regarding His-bundle pacing include more complex device programming (which requires a deep understanding from every person who is likely to interrogate or reprogram the device) as well as the unknown effects of a long-term (ie, several decades-long) His-bundle lead position and the inevitable extraction of these leads.

Despite these concerns, the likely benefits of His-bundle pacing are great enough that the adoption of this technique will continue to expand. This growth will likely be bolstered by improved tools, such as deflectable His-bundle delivery catheters or catheters of various shapes (such as is seen with coronary angiography). Therefore, as with many other rapidly evolving procedural techniques, trial data may underestimate future benefits due to limitations of the technology available at the time of enrollment.

While we expect to see an escalation in His-bundle pacing over time, we also expect an increase in leadless pacing options and fully subcutaneous defibrillators. It remains to be identified how these three technologies will interface with one another, though the His-bundle region does not seem to be well-suited to currently available leadless pacing systems due to the lack of trabeculations. Therefore, while the immediate future of His-bundle pacing seems poised to leap forward with small changes in the delivery system, the more distant future of pacing in general is not currently clear at this time.
